# Clinical Genetics of Inherited Arrhythmogenic Disease in the Pediatric Population

**DOI:** 10.3390/biomedicines10010106

**Published:** 2022-01-05

**Authors:** Estefanía Martínez-Barrios, Sergi Cesar, José Cruzalegui, Clara Hernandez, Elena Arbelo, Victoria Fiol, Josep Brugada, Ramon Brugada, Oscar Campuzano, Georgia Sarquella-Brugada

**Affiliations:** 1 Arrhythmias Unit, Hospital Sant Joan de Déu, University of Barcelona, 08007 Barcelona, Spain; estefania.martinez@sjd.es (E.M.-B.); sergio.cesar@sjd.es (S.C.); josecarlos.cruzalegui@sjd.es (J.C.); clara.hernandez@sjd.es (C.H.); victoria.fiol@sjd.es (V.F.); jbrugada@clinic.cat (J.B.); 2 Centro de Investigación Biomédica en Red, Enfermedades Cardiovasculares (CIBERCV), 28029 Madrid, Spain; EARBELO@clinic.cat (E.A.); rbrugada@idibgi.org (R.B.); 3 Arrhythmias Unit, Hospital Clinic, University of Barcelona-IDIBAPS, 08036 Barcelona, Spain; 4 Medical Science Department, School of Medicine, University of Girona, 17004 Girona, Spain; 5 Cardiovascular Genetics Center, University of Girona-IDIBGI, 17190 Girona, Spain; 6 Cardiology Service, Hospital Josep Trueta, University of Girona, 17007 Girona, Spain

**Keywords:** Brugada syndrome, catecholaminergic polymorphic ventricular tachycardia, channelopathies, long QT syndrome, short QT syndrome

## Abstract

Sudden death is a rare event in the pediatric population but with a social shock due to its presentation as the first symptom in previously healthy children. Comprehensive autopsy in pediatric cases identify an inconclusive cause in 40–50% of cases. In such cases, a diagnosis of sudden arrhythmic death syndrome is suggested as the main potential cause of death. Molecular autopsy identifies nearly 30% of cases under 16 years of age carrying a pathogenic/potentially pathogenic alteration in genes associated with any inherited arrhythmogenic disease. In the last few years, despite the increasing rate of post-mortem genetic diagnosis, many families still remain without a conclusive genetic cause of the unexpected death. Current challenges in genetic diagnosis are the establishment of a correct genotype–phenotype association between genes and inherited arrhythmogenic disease, as well as the classification of variants of uncertain significance. In this review, we provide an update on the state of the art in the genetic diagnosis of inherited arrhythmogenic disease in the pediatric population. We focus on emerging publications on gene curation for genotype–phenotype associations, cases of genetic overlap and advances in the classification of variants of uncertain significance. Our goal is to facilitate the translation of genetic diagnosis to the clinical area, helping risk stratification, treatment and the genetic counselling of families.

## 1. Introduction

While sudden cardiac death (SCD) is a rare event in pediatrics, it has a significant social impact, since it often presents as the first symptom in previously healthy children. The reported incidence rate of SCD in children and young adults is estimated to be between 1.3 and 1.7 per 100,000 persons-year [1,2], with twice as many cases in males than females. SCD is almost 10 times higher in young adults aged 31–35 years, while very low in children aged 6–10 years of age [3]. In addition to age and sex, other demographic factors such as ethnicity, exercise habits and geographic location impact on the incidence and survival rate of SCD, but the causes remain to be established [4,5]. During the first year of life, unexpected deaths remain unexplained after a comprehensive autopsy accounts for the highest infant mortality rate and is usually labelled as sudden infant death syndrome (SIDS) [6].

The most prevalent etiologies identified by medico-legal autopsy in children and young adults are cardiomyopathies, accounting for about 30% of SCD [7]. Nevertheless, a negative autopsy result is the most common finding in pediatrics, accounting for 40–50% of cases in the population under 16 years old. This outcome suggests a diagnosis of sudden arrhythmic death syndrome (SADS), the leading cause of SCD in children [8]. In SADS, the regular heart rhythm is abruptly replaced by a lethal ventricular arrhythmia, in the context of a structurally normal heart [9], the so-called inherited arrhythmia syndromes (IASs) or cardiac channelopathies.

Thanks to recent technological advances in the field of genetics, a considerable number of potentially IASs-causing genes have been identified although not comprehensively characterized. Current panels used in genetic testing of IASs range from the most common genes recommended by the clinical guidelines to the less common genes described in a few families, in large part not definitively associated with any IASs. Those that can be tested in a fast and cost-effective approach in clinical practice remain a current matter of argument. Therefore, studying a larger number of genes represents new challenges, such as limitations in establishing valid associations between genes and phenotypes. To address this problem, in 2013 the National Institute of Health (NIH) encouraged the development of ClinGen (Clinical Genome Resource https://clinicalgenome.org/ accessed on 3 December 2021), an international consortium of geneticists, genomic scientists and experts in the clinical field, which has established an evidence-based gene curation approach to establish gene–disease associations. Recently, evaluations of IAS-associated genes according to ClinGen’s approach have been published [10,11,12,13]. A further major challenge is due to the large number of variants of uncertain significance (VUS) yielded in next-generation sequencing (NGS) studies. The American College of Medical Genetics and Genomics/Association for Molecular Pathology (ACMG/AMP) guidelines are the current gold standard for classifying genetic variants. However, these guidelines were designed mainly for the classification of genetic variants in recessive or dominant pathologies with complete penetrance. IASs are characterized as diseases with an autosomal dominant inheritance pattern (AD), incomplete penetrance and variable expressivity, thus some criteria of the ACMG/AMP guidelines present limitations for their classification. Together with its strict criteria, this leads to high rates of VUS in the genetic diagnosis of IASs [14,15,16,17], making it necessary to work with adjusted ACMG/AMP criteria for each pathology and gene. Some researchers in the field of primary arrhythmias have aimed to perform a quantitative implementation of the ACMG/AMP guidelines for IAS’s genetic testing, with success in reducing the VUS rate [18].

We provide a state of the art overview of the genetic diagnosis of IASs in the pediatric population and its translation into clinical practice, based on international expert guidelines, recent advances in evidence-based genetic curation, and IAS-focused variant classification. We will emphasize genotype–phenotype and variant–phenotype correlation. Our goal is to facilitate translation of the genetic diagnosis to the clinical area, helping in risk stratification, treatment and the genetic counseling of families.

## 2. Inherited Arrhythmia Syndromes

Cardiac channelopathies are caused by defects in the genes encoding sodium (Na^+^), potassium (K^+^) and calcium (Ca^2+^) ion channels or their associated proteins. Defects in these proteins impair the generation and transmission of the action potential (AP) predisposing the patient to fatal arrhythmias [19]. The arrhythmias are usually of the type ventricular tachycardia (VT) or rapid ventricular fibrillation (VF), and generally polymorphic [20]. Despite the fact that each channelopathy usually has a characteristic electrocardiogram (ECG) profile, an associated clinical phenotype and specific genes involved, these factors can often be shared by two or more syndromes, leading to difficulties in establishing a differential diagnosis. In addition, some of these syndromes are not associated with baseline ECG abnormalities, which make them difficult to diagnose, with the added distress that SCD or resuscitated cardiac arrest is the initial symptom in most cases [21]. Therefore, an accurate genetic diagnosis can help to predict the prognosis and management of patients and their families. IASs are predominantly monogenic syndromes with an AD inheritance pattern, characterized by incomplete penetrance and variable expressivity [22]. Phenotypes with an autosomal recessive (AR) and X-linked inheritance pattern occur, but they are a minority [23]. Four major IASs can be typically observed in pediatrics: long QT syndrome (LQTS), short QT syndrome (SQTS), Brugada syndrome (BrS) and catecholaminergic polymorphic ventricular tachycardia (CPVT) [24].

## 3. Long QT Syndrome

LQTS is the most common of the IASs and the main contributor for SCD in young people under 20 years of age. LQTS affects approximately 1 per 2000–2500 persons-year [25] with a slight predominance of females [26]. Moreover, it is the major arrhythmogenic syndrome, responsible for infants’ deaths, especially in the first days of life [27]. It is characterized by QT interval prolongation and T-wave abnormalities in ECG, polymorphic ventricular tachycardia (PVT) in *torsade de pointes* (TdP), VF and syncope or SCD [28]. LQTS can be hereditary, congenital or acquired, usually associated with drugs and electrolyte imbalance (hypokalemia) (http://www.torsades.org accessed on 3 December 2021).

### 3.1. Genetics

Originally characterized as an AD inheritance syndrome, called Romano–Ward syndrome (LQT1–6 and LQT9–13), today 17 LQTS-associated genes are known (*AKAP9*, *ANK2*, *CACNA1C*, *CALM1*, *CALM2*, *CAV3*, *KCNE1*, *KCNE2*, *KCNH2*, *KCNJ2*, *KCNJ5*, *KCNQ1*, *SCN1B*, *SCN4B*, *SCN5A*, *SNTA1* and *TRDN*) (Table 1) [29,30]. Most manifest with an AD inheritance pattern, except for the Jervell and Lange–Nielsen syndromes (JLNS) [31] and the recently characterized triadin knock out syndrome (LQT17) [32], which are inherited in an AR manner. In the most recent evaluation by an expert consensus, only three of these genes were considered to have adequate evidence to be classified as definitive for typical LQTS (*KCNQ1*, *KCNH2*, *SCN5A*) and four genes for LQTS with atypical features (*CALM1*, *CALM2*, *CALM3*, *TRDN*). The remaining 10 genes, despite being associated with LQTS, were considered to require further evidence for their classification as causals [10].

### 3.2. Definitive Genes for LQTS

Pathogenic variants in 3 genes (*KCNQ1*, *KCNH2* and *SCN5A*) are responsible for approximately 90% of all diagnosed cases of LQTS [98]. With LQT1 (*KCNQ1*) accounting for 30–35% of cases, LQT2 (*KCNH2*) between 25–30% and LQT3 (*SCN5A*) around 5–10% [99,100]. The age of onset of clinical manifestations in each type is variable. LQT1 affect predominantly the pediatric population between 5–15 years of age, whereas LQT2 and LQT3 occur during puberty or later [34,101]. Even though severe causes of neonatal or even fetal manifestation have been reported, a similar mechanism for arrhythmogenesis is found in types 1 and 2. Both syndromes are caused by loss-of-function pathogenic variants in genes encoding for K^+^ channels. Disruption of these channels causes a repolarization delay with an increase in the AP (phase 3), leading to the QT interval prolongation. In comparison, in LQT3, this phenotype is caused by gain-of-function pathogenic variants in the *SCN5A* gene, which codes for a Na^+^ channel [102]. Adrenergic stimuli have an important role as a trigger of symptoms in LQTS types 1 and 2. Physical exercise is the main trigger of SCD in LQT1. A strong sudden startle, loud noise or emotional stress are triggers of LQT2. Moreover, LQT3 presents with malignant arrhythmias at rest or during sleep [34].

### 3.3. Definitive Genes for LQTS with Atypical Characteristics

Loss-of-function pathogenic variants in the *CALM1*, *CALM2*, *CALM3* and *TRDN* genes cause LQTS types 14 to 17, respectively. The *CALM*, *CALM2* and *CALM3* genes code for calmodulin, and the *TRDN* gene for triadin, proteins involved in calcium-dependent processes and ion channel regulation [43,46]. LQT14–16 present atypical features, including seizures and neurodevelopmental delay [103] with symptoms manifesting in infants and young children between the ages of 0–5 years old and a high mortality rate [104]. LQT17 or triadin knockout syndrome (TKOS) also has poor prognosis, and exercise-induced SCD occurs in children aged 0–3 years old. Almost all patients are symptomatic by the age of 10 years old. The observation of negative T waves in the precordial leads is characteristic of LQT17 [47,105].

### 3.4. Genes with Moderate or Limited Evidence for LQTS

According to the recent evaluation by Adler et al., there is insufficient evidence to classify 10 of the 17 LQTS-related genes (*CACNA1C*, *AKAP9*, *ANK2*, *CAV3*, *KCNE1*, *KCNE2*, *KCNJ2*, *KCNJ5*, *SCN4* and *SNTA1*) as LQTS-causing genes [31983240]. Each of these types represents less than 1% of LQTS. Some of them are associated with phenotypic syndromes and specific ECG features (Table 1). These include Andersen–Tawil syndrome (LQT7, *KCNJ2*) in which skeletal developmental abnormalities are observed [54]; Timothy syndrome (LQT8, *CACNA1C*) with characteristic neurological, facial and limb features [106] and JLNS syndrome, associated with sensorineural deafness (*KCNQ1-KCNE1* genes) [107]. Although the association of pathogenic variants in these genes with multiorganic syndromes is clear, the level of evidence for the specific cardiac phenotype is not so clear, and further studies are needed [10]. 

### 3.5. Genetic Modifiers and Acquired LQTS

GWAS studies have identified polymorphisms associated with increased risk of triggering LQTS [108,109]. Common variants in the *NOS1AP* (Nitric Oxide Synthase 1 Adaptor Protein) gene confer an increased risk of SCD in patients with LQT1 [110]. Likewise, some variants have a protective effect, as is the case of p.H558R (*SCN5A*), which reduces the pathogenic effect of other pathogenic variants, producing a less severe phenotype [22]. The common variants p.D85N (*KCNE1*) and T8A-MiRP1 (*KCNE2*) in Caucasians [111,112] and p.S1103Y (*SCN5A*) in African Americans [113], confer risk in the presence of other triggers such as drugs, a phenotype known as acquired LQTS (aLQTS). These variants are insufficient by themselves to cause LQTS in the absence of other interval-prolonging factors [10,114]. However, they are of major importance due to the high frequency of aLQTS [115].

### 3.6. Diagnosis

According to the 2015 ESC Guideline, LQTS is diagnosed when the patient presents one of the following criteria: a corrected QT (QTc) ≥480 ms (repeated 12-lead ECGs), a LQTS risk score >3, or when a pathogenic alteration in one of the LQTS-causing genes is identified [116]. The LQTS risk score combines the altered ECG parameters with the patient’s clinical and family history and is based on the clinical score proposed by Schwartz in 1993 [117]. Regardless, in the presence of unexplained syncope a QTc ≥460 ms is sufficient to diagnose LQTS [116] [26318695]. LQTS presents cardiac and extracardiac phenotypic features, as well as ECG characteristics that allow its classification, although 25% of individuals with positive genetics show a normal baseline ECG [118].

### 3.7. Risk Stratification

Multiple factors are known to raise the likelihood of SCD in patients with LQTS. The presence of a QTc >500 ms is the strongest of these predictors [119,120]. In children, this parameter can be modified by the patient’s age, sex and genotype, with a critical transition period between 12–14 years of age [121]. Patients who have suffered syncope during childhood have an increased risk of recurrent episodes, which can be reduced through the use of beta-blockers (BB) and/or implantable cardiac defibrillators (ICD) [122,123]. Among the types, LQT3 has a worse prognosis and the first presentation is usually SCD [119,124]. Likewise, LQT14–16 types have been associated with a very severe phenotype in infants and poor response to available therapies [103]. Women with LQT2 have an increased risk of SCD in the first 6 months postpartum, suggesting a potential hormonal effect [125]. Pathogenic variants type and location, as well as other additive genetic factors may increase the risk of SCD. For example, female adults with LQT2 run a higher risk of SCD than males. However, when missense pathogenic variants in the *KCNH2* gene are in the pore loop regions, males are at a higher risk of SCD than their female counterparts [126]. Indeed, pathogenic variants in these regions have been found to be associated with QT interval prolongation and the development of TdP during fever, suggesting that fever may be a potential trigger of arrhythmias in patients with LQT2 [127].

### 3.8. Genetic Counselling

LQTS has incomplete penetrance and variable expressivity, even in the same family. The penetrance is estimated to be about 40%, a range that can vary depending on genotype, pathogenic variants type and location, age and sex, among other things [22,128]. The identification of a pathogenic variant in one of the LQTS-associated genes (Table 1) is important to establish a differential diagnosis of patients and their relatives. In LQTS, treatment and risk stratification differ depending on the gene causing the disease, and can be variant-specific [129]. According to guideline recommendations, only genes with definitive evidence for LQTS (*KCNQ1*, *KCNH2* and *SCN5A*) should be routinely used in the evaluation of patients and their families. In patients with clinical findings consistent with the phenotypic expression demonstrated in LQTS with atypical features, related genes (*CALM1*, *CALM2*, *CALM3* and *TRDN*) should also be tested [10]. Genetic testing can identify an LQTS-causing alteration in 70–80% of cases [99]. The proportion of LQTS caused by de novo pathogenic variants is difficult to estimate, but is expected to be low [130]. Between 5–9% of familial cases of LQTS have two or more pathogenic variants (biallelic or digenic), which have been associated with a more severe phenotype [131,132]. The coexistence of two or more pathogenic variants could explain the variable expressivity observed in some families [133]. The copy number variants (CNV) detection rate among LQTS families is not very clear, but is estimated to be between 2–11% [134,135,136,137,138]. Population screening by ECG has been promoted to identify individuals at risk of LQTS, which has been successful in reducing SCD rates among patient family members [139] neonates [27,140] and athletes [141,142].

### 3.9. Management and Treatment

In children with LQTS the first approach is to avoid genotype-specific triggers, such as competitive sports, especially swimming in LQT1, and exposure to loud noise in children with LQT2 as well as the avoidance of the QT interval prolonging drugs in all carriers of LQTS-associated variants (http://crediblemeds.org/ accessed on 3 December 2021) [116]. Long-acting BB (nadolol) are recommended in all types, including asymptomatic genetic carriers [143,144], as their use decreases the risk of SCD [145]. Specific treatment of LQT3 with mexiletine and/or flecainide has proven to be highly effective. In LQT1 the use of BB is very effective, and some authors suggest that it is not necessary to place an ICD in patients at low risk of SCD (asymptomatic prepubertal girls and adults >20 years with normal ECG) [146]. Left-cardiac sympathetic denervation is indicated in patients with LQT1 or when BB therapy is contraindicated or badly tolerated [147]. ICDs are used in patients at high risk of SCD despite previous therapies, in those who have previously presented syncope while taking BB, and effective LCSD (left cardiac sympathetic denervation) has been performed [148]. Despite its efficacy, ICD has a high economic cost and can present numerous complications. Its use in the pediatric population should be assessed on a case-by-case basis by specialists [149].

## 4. Brugada Syndrome

BrS in children and young adults is rare, and its incidence rate and clinical implications remain unclear. In the general population, its prevalence is estimated to be between 1 in 2000–5000 person-years [150]. The syndrome has a higher prevalence in Southeast Asian countries [151], and is more frequent among males than females [152]. It is characterized by a right bundle branch block, a very sharp T wave and spontaneous or drug-induced ST-segment elevation (J point) in the right precordial leads (V1–V3), known as ‘type-1’ BrS ECG pattern [64]. Clinical manifestations may appear between the ages of 2 months and 77 years old, but the mean age of presentation is 40 years old [153]. Symptoms present at rest, during sleep or febrile episodes, including nocturnal agonal respirations, palpitations, seizures, and polymorphic ventricular tachycardia (PVT) or VF. Most individuals remain asymptomatic, although SCD occurs in 17–42% of the cases and can be the initial presentation [154].

### 4.1. Genetics

Genetically described as a Mendelian syndrome with an autosomal dominant inheritance pattern and incomplete penetrance [155], recent evidence suggests that BrS may be an oligogenic disease, involving several genetic factors [156]. However, the lack of conclusive data on these genetic alterations leads it to remain classified as a monogenic syndrome [157]. To date, more than 20 genes have been associated with BrS (*ABCC9*, *ANK2*, *CACNA1C*, *CACNA2D1*, *CACNB2*, *GPD1-L*, *HCN4*, *KCND3*, *KCNE3*, *KCNE5*, *KCNH2*, *KCNJ8*, *PKP2*, *RANGRF*, *SCN10A*, *SCN1B*, *SCN5A*, *SCN2B*, *SLMAP* and *TRPM4*), reappraisal of these genes by Hosseini et al., established that only *SCN5A* had definitive evidence of being a causal gene [12] and its genetic analysis is the only one recommended by current guidelines [23,158]. 

### 4.2. Definitive Gene for BrS

Loss-of-function pathogenic variants in the *SCN5A* gene account for approximately 30% of genetically positive BrS cases. This gene encodes the alpha subunit of the cardiac sodium channel Nav1.5, responsible for phase 0 of the AP. Inactivation of the channel leads to a delay in ventricular polarization, resulting in the development of ventricular tachycardia and fibrillation (VT/VF) [150].

### 4.3. BrS2–12 and Other Susceptibility Genes with Limited Evidence 

Pathogenic variants associated with BrS2–12, genes (*GPD1-L*, *CACNA1C*, *CACNB2*, *SCN1B*, *KCNE3*, *SCN3B*, *HCN4*, *KCND3*, *KCNJ8*, *CACNA2D1* and *MOG1*) together represent less than 5% of all diagnosed cases [159]. Over the past few years, other genes have been suggested as possible causes of BrS (*ABCC9*, *ANK2*, *FGF12*, *HEY2*, *KCND2*, *KCNH2*, *KCNE5*, *LRRC10*, *SEMA3A*, *PKP2*, *RANGRF*, *SCN10A*, *SCN2B*, *SLMAP* and *TRPM4*), but no comprehensive clinical and cellular studies have confirmed this association [157]. All of them follow an AD inheritance pattern, except for the *KCNE5* gene, which follows an X-linked dominant pattern [160,161]. 

### 4.4. Diagnosis

According to the 2015 ESC guidelines, BrS is diagnosed in patients with a ‘type 1’ ECG pattern, ST-segment elevation ≥2 mm (J-point) in one or more of the right precordial leads (V1–V3) [116]. This ECG pattern may occur spontaneously or be unmasked by a provocation test with a class Ic drug (sodium channel blockers such as ajmaline, flecainide, procainamide, or pilsicainide), in this case additional clinical criteria are required for diagnosis [64]. Fever is a trigger for ventricular arrhythmias in patients with BrS and may unmask the characteristic ECG pattern, especially in children under 5 years of age [162]. A 12-lead ECG is recommended during febrile episodes in children with a family history of BrS and in all children with febrile seizures [163]. Initially presumed not to have any structural abnormalities, postmortem histological studies and endomyocardial biopsies have shown changes at the tissular and molecular level in patients with BrS. These changes include localized electroanatomical and structural abnormalities in the right ventricular outflow tract (RVOT), fibrosis, fatty infiltration, increased epicardial collagen, and decreased expression of Connexin 43 at right ventricular gap junctions [164,165,166].

### 4.5. Risk Stratification

Despite advances in risk stratification of IAS, in BrS it remains challenging. The most important risk marker is the presentation of a previous arrhythmogenic event (AE, AF, syncope, or SCD), which increases the likelihood of SCD in both young and adults [167,168]. The age of onset is a notable prognostic marker. For instance, although BrS is uncommon in children, they present with a more severe form of the disease [169]. Gender is an important risk factor, with males being up to 5–8 times more affected than females, presenting a more severe phenotype, earlier symptom debut and a higher number of events [152,170]. These gender differences have not been observed in children under 12 years old [167]. Family history of SCD and the presence of pathogenic variants in *SCN5A* could also be predictors of high risk in adults and adolescents, [167] even though their role in risk stratification is still controversial [171,172]. Other risk factors observed are spontaneous variation of the ‘type 1’ ECG pattern and fragmentation of the QRS complex [171,173], nevertheless further studies are required to confirm this association.

### 4.6. Management and Treatment

In children with BrS or a family history, BrS-inducing drugs should be avoided (http://www.brugadadrugs.org accessed on 3 December 2021). Appropriate treatment of any fever with antipyretic drugs should be provided [167]. ICD implantation is the only treatment that reduces the risk of SCD in BrS. It is indicated in all patients resuscitated from arrhythmic syncope, those with documented VT or VF or with a spontaneous ‘type 1’ ECG pattern [116,149]. In patients with electrical storms, administration of isoproterenol is recommended [174,175]. Quinidine is recommended for patients who refuse ICD implantation or in those who, despite having an ICD, still have a high risk of SCD [176,177]. Catheter ablation is useful in high-risk patients with a history of electrical storms or repeated appropriate ICD shocks [178]. ICD implantation in asymptomatic individuals with a ‘type 1’ ECG pattern for primary prevention, including children, remains controversial and is a challenge for specialists who have to handle it on a case-by-case basis [149,177].

### 4.7. Genetic Counseling

BrS penetrance is highly variable in the different published studies. It is estimated to range from 12.5% to 50% [22,179]. About 70–80% of families with BrS do not have a genetic diagnosis. However, although the results of genetic screening do not currently influence prognosis or treatment, genetic testing should be performed in all first-degree relatives if the index case tested positive [99,116]. Moreover, the ECG pattern of BrS ‘type 1’ is uncommon in children and genetic testing may help with their diagnosis [21]. In BrS families counseling should also include an ECG, because negative-genotype positive-phenotype cases are not uncommon [180]. The proportion of cases caused by de novo pathogenic variants is estimated at 1% and the number of cases with a CNV variant is approximately 1.3–2.9% [138,181]. ECG screening using a provocation test for BrS detection is controversial [182,183], but should be performed when an abnormal ECG is found. ECG screening has benefits in the prevention of SCD in neonates [140] and the young population [184]. 

## 5. Short QT Syndrome

SQTS is an extremely rare inherited disease associated with SCD. To date, less than 200 cases have been reported worldwide [93]. The estimated prevalence varies between 0.18–2.9%, with a higher incidence in males than females [25]. The incidence rate can even be lower (0.02–0.10%) if more restrictive values are considered for its diagnosis [185,186]. While prevalence of the syndrome in children and adolescents is low (about 0.05%), early detection is important, as it is potentially lethal for all age groups. It is characterized by a short QT interval on the ECG (<330 ms), with an asymmetric and peaked T wave. Symptoms occur mostly in men between the ages of 14 and 40 and may be favored by hormonal causes [187]. Cardiac events usually occur in adrenergic situations (noise or exercise), although it can also occur at rest. Clinical presentation includes ventricular repolarization abnormalities (AF and VT) and syncope. The probability of presenting SCD as the first symptom increases with age, reaching 41% at the age of 40 [188]. Currently, approximately 40% of cases remain asymptomatic [189].

### 5.1. Genetics

At present, nine genes have been associated with SQTS (*CACNA1C*, *CACNA2D1*, *CACNB2*, *KCNH2*, *KCNJ2 and KCNQ1*, *SLC22A5*, *SLC4A3* and *SCN5A*) [190]. Evaluation of these genes, by Walsh et al., showed that only the *KCNH2* gene had definitive evidence for SQTS causality. Three other genes (*KCNQ1*, *KCNJ2*, *SLC4A3*) presented strong to moderate evidence. Causality of the other SQTS-associated genes remains still in dispute [11]. These data are consistent with the findings published by Campuzano et al., who found that all variants with a conclusive pathogenic role in SQTS clustered in three genes (*KCNQ1*, *KCNH2* and *KCNJ2*). In that study, the *SLC4A3* gene was excluded, since carriers were in a gray zone of SQTS diagnosis (with a QTc ≤370 ms) [190].

### 5.2. SQT1 Definitive Gene: KCNH2

The p.T618I and p.N588K pathogenic variants in the *KCNH2* gene are the most frequent associated with SQTS, accounting for 85% of SQT1 and 55% of all genetically identified cases of SQTS [189]. Gain-of-function pathogenic variants in *KCNH2* lead to prolonged K^+^ channel activation and accelerated cardiac repolarization with shorter refractory periods, potentially triggering life-threatening supraventricular and ventricular arrhythmias [189].

### 5.3. Genes with Strong or Moderate Evidence for SQTS

SQT2 and SQT3 are driven by gain-of-function pathogenic variants in genes encoding for K^+^ channels (*KCNQ1* and *KCNJ2*, respectively). The mechanism of arrhythmogenicity is similar to that presented by *KCNH2*. The *SLC4A3* gene, recently associated with SQTS, presents an uncommon mechanism for the development of malignant arrhythmia. *SLC4A3* encodes the plasma membrane anion exchange protein 3 (AE3) and acts by mediating part of the Cl^−^/HCO3^−^ exchange in cardiac myocytes. Loss-of-function pathogenic variants in the *SLC4A3* gene would cause an increase in pH_i_ and a decrease in [Cl^−^]_i_, shortening the duration of the AP [94].

### 5.4. Diagnosis

According to the 2015 ESC guidelines, SQTS is diagnosed by the presence of a QTc ≤340 ms, or ≤360 ms when one the following clinical criteria occur: the detection of a known pathogenic alteration, a family history of SQTS, a family history of SCD before the age of 40 years, or reanimated cardiac arrest with a structurally normal heart [116]. 

### 5.5. Risk Stratification

On account of the limited number of patients with SQTS and the phenotypic variability of the syndrome, risk stratification currently represents a challenge. To date, the only predictor of SCD found in patients with SQTS is a history of cardiac arrest [93]. Genotype-phenotype correlation studies have found that SQTS1 manifests at an older age and patients have a shorter QTc than other patients with SQTS. Nevertheless, no association of this reduction with an increased risk of SCD has been found [191].

### 5.6. Management and Treatment

ICD implantation is recommended for all patients with SQTS, especially for patients who have survived an aborted cardiac arrest or have presented spontaneous sustained VT [186,192]. QT interval prolonging drugs (quinidine and sotalol) should be considered for all patients at risk of SQTS in both, asymptomatic and symptomatic patients who don’t have an ICD, especially young children [186]. 

### 5.7. Genetic Counselling

Due to the low number of cases, the penetrance of SQTS is difficult to estimate. However, pathogenic variants with a penetrance of 100% have been reported [189]. The diagnostic yield of genetic testing in SQTS is low (<25%) [186]. Current guidelines recommend analysis of five genes: *KCNH2*, *KCNQ1*, *KCNJ2*, *KCNJ2*, *CACNA1C* and *CACNB2* in the diagnosis of SQTS [116], with the *KCNH2* gene as the most cost-effective option [193]. Familial genetic analysis is recommended, both to clarify the pathogenic role of newly identified variants and to identify family members at risk for SCD. To date, there is no published data on CNV analysis on patients with SQTS. De novo variants in the *KCNQ1* gene have been associated with a particular in utero phenotype with clinical diagnosis of AF with concomitant bradycardia and short QT interval [194,195]. Some researchers support the screening of SQTS in the pediatric population, given its high lethality and the benefits of early diagnosis in the prevention of SCD. These studies have shown that the diagnostic criteria for QTc should be adjusted in each population based on factors including sex and age, to avoid false positives [196,197,198,199].

## 6. Catecholaminergic Polymorphic Ventricular Tachycardia

The prevalence of CPVT is estimated to be 1 per 10,000 population. However, the real prevalence is uncertain, as it might be underestimated due to its high lethality at a young age and difficulty in diagnosis [25,200]. CPVT is characterized by a bidirectional polymorphic VT, triggered by an adrenergic stimulus mainly during exertion, extreme stress or emotion that can lead to syncope and SCD. Syncopal episodes are increasingly reported during “awake rest” possibly due to anxiety, stress, or other psychological stimuli unrelated to exertion [201]. The age of onset can range from infancy to the age of 30, although it is more common in children aged 7–10 years old [202]. By the age of 10, about 35% of patients are symptomatic, increasing to 72% by the age of 21 [203]. A younger age of debut is often accompanied by more severe phenotypes and increased risk of SCD [42]. 

### 6.1. Genetics

According to the recent evaluation by Walsh et al., seven genes were classified as causing of CPVT with definite to moderate evidence. Four of them present an AD inheritance pattern (*RYR2*, *CALM1*, *CALM2*, *CALM3*) and three AR inheritance (*CASQ2*, *TRDN*, *TECRL*). Three genes (*KCNJ2*, *PKP2*, *SCN5A*) were reported for phenotypes that were not representative of CPVT, while the reported variants in the *ANK2* gene were considered too common in the population to be disease-causing (Table 1) [11]. 

### 6.2. Definitive Genes for CPVT

CPVT1 is the most prevalent variant, accounting for more than 60% of all genetically diagnosed cases of CPVT [204]. CPVT1 with a high incidence in children around 10 years of age [205]. Thus, it is caused by pathogenic variants in the *RYR2* gene, which encodes for ryanodine receptor 2, responsible for calcium regulation in the cardiomyocyte. A majority of pathogenic variants in the *RYR2* gene are gain-of-function, which promote an increased Ca^2+^ release from the sarcoplasmic reticulum of cardiomyocytes into the cytoplasm leading to late after-depolarizations [48]. The remaining types of CPVT represent about 10% of cases. Most are caused by loss-of-function pathogenic variants in genes encoding proteins involved in the storage and release of Ca^2+^ in the sarcoplasmic reticulum [206]. CPVT2 is caused by homozygous or compound heterozygous pathogenic variants in the *CASQ2* gene, following an AR inheritance model. CPVT2 accounts for about 3–5% of cases [203], affecting mainly children around the age of 7 years [205]. Pathogenic variants in *TRDN*, *CALM1* and *TECRL* are responsible for CPVT3, CPVT4, and CPVT5, respectively. Each represents about 1–2% of the cases. CPVT3 and CPVT5 follow an AR inheritance pattern, even though AD inheritance has also been observed in some families [42,91]. In contrast, CPVT5 is presented with an AD inheritance pattern, a younger age of onset (approximately 2.3 years) and more severe phenotypes, being highly lethal in children [205,207]. Along with *TRDN* and *CALM1*, gain-of-function pathogenic variants in the *CALM2* and *CALM3* genes have been associated with the development of atypical CPVT, presenting more complex and variable associated phenotypes than classic [208,209].

### 6.3. Diagnosis

CPVT is diagnosed when exercise- or emotion-induced bidirectional or polymorphic VT is detected, in the presence of a structurally normal heart or in patients carrying pathogenic alterations in the definitive genes for CPVT [200]. Since the resting ECG is usually normal, ECG during exercise and Holter monitoring play a relevant role in the diagnosis. The disease can be easily missed or misdiagnosed; for instance, many children are initially diagnosed with epilepsy, as syncope may be associated with seizure movements.

### 6.4. Risk Stratification

Between 30–50% of patients with CPVT will experience SCD before the age of 30 [210,211]. The event rate in untreated children under 8 years old has been estimated at 58% that can be reduced to 27% with adherence to BB treatments [203]. *RYR2* is one of the most prevalent genes in cohorts of patients with unexplained SCD, occurring in 5–10% of cases [212]. While the variable expressivity of the CPVT phenotype could be explained by the influence of other genetic and non-genetic factors, no genetic modifiers have been identified in CPVT to date [108,213]. Patients with the recessive form of CPVT and a younger age at diagnosis have a more severe phenotype [48,203]. The location of the rare variant may be a possible disease modifier [201]. No gender- or age-dependent differences in arrhythmic risk in children have been found to date [214]. However, further studies in CPVT risk stratification are needed to draw definitive conclusions. 

### 6.5. Management and Treatment

In children with CPVT, avoidance of phenotype triggers such as competitive sports, strenuous exercise (especially swimming), and stressful environments is recommended. BB are the first-line of treatment, their use is recommended in all patients, even in genetically identified asymptomatic patients [116]. About 25% of children experience syncope or cardiac arrest despite treatment with BB [215]. In these patients, it is advisable to include flecainide therapy and/or left cardiac sympathetic denervation (LCSD). LCSD has been proven to reduce the rate of arrhythmic events in patients with LQTS and CPVT [200,216]. It should be noted that ICD therapy may be counterproductive in CPVT, because the discharge may activate adrenergic production and exacerbate the VT storm, so its implantation should be assessed by the specialist [217].

### 6.6. Genetic Counseling

CPVT penetrance can vary between 63–78% [22,213,218]. The diagnostic yield is high, with a positive genetic result in 60–65% of the studied cases [25]. Genetic testing is recommended by expert consensus, with *RYR2* and *CASQ2* genes as the most cost-effective options [23]. Identification of family members at risk is critical to avoid SCD, which is the first manifestation in up to 30–50% of cases [210]. CPVT has a high incidence of de novo variants, which are found in approximately 50% of genetically diagnosed CPVT patients [25]. CNV in *RYR2* have been associated with CPVT [138,219,220], while the other CPVT-related genes have not been examined so far.

## 7. Genetic Overlap

**Figure 1:**
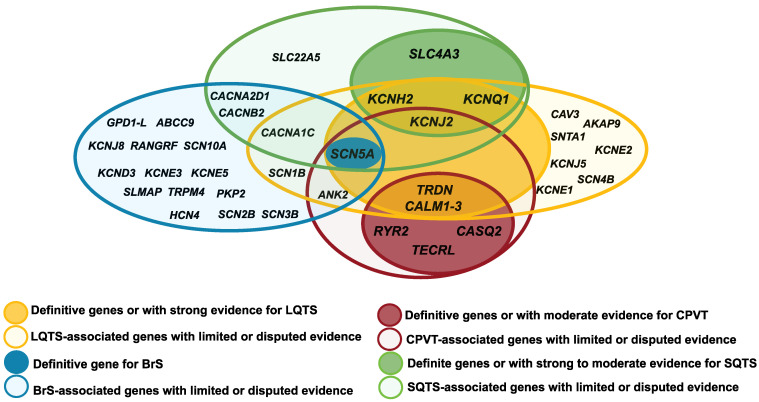
Diagram of the overlap between the genes IASs-associated genes: Brugada syndrome (BrS); short QT syndrome (SQTS); long short QT syndrome (LQTS) and catecholaminergic polymorphic ventricular tachycardia (CPVT).

Several causative genes for IASs are common to two or more syndromes (LQTS, SQTS, BrS, and CPVT) (Figure 1) and have been found to overlap with other cardiac and extracardiac clinical phenotypes (Table 1). This genetic overlap could be explained since pathogenic variants can alter different properties of the channels or proteins, affecting ion exchange, interaction with auxiliary proteins, as well as gene expression. The final phenotype would depend not only on which property is affected, but also how it is affected. Notably, some genes that cause LQTS are the same genes that also cause SQTS (*KCNQ1*, *KCNH2*, *KCNJ2*, *CACNA1C*, *CACNB2*, and *CACNA2D1*), CPVT (*CALM1*, *CALM2*, *CALM3*, *TRDN*, *KCNJ2*, *SCN5A*, *ANK2* and *TECRL*) or BrS (*SCN5A*) [42,89]. However, the pathogenic variants usually cause an opposite effect on the channel.

Some pathogenic variants have been found to lead to complex overlapping forms of two or more syndromes. Several publications support the fact that pathogenic variants in *KCNQ1*, *ANK2*, *KCNE1*, *KCNE2*, *KCNH2*, *KCNJ2* and *SCN5A* can lead to a complex overlapping phenotype of LQTS, CPVT and ventricular ectopy [42,48,92]. Hirose et al., recently described in a cohort of children (<16 years old) the association of loss-of-function pathogenic variants in *RYR2* with various types of arrhythmia, including LQTS, VF and scTdP, depending on the alteration of channel activity [83,84]. The pathogenic variants p.I4855M and deletion of exon 3 in the *RYR2* gene have been associated with the rare syndrome of left ventricular non-compaction (LVNC) overlap and CPVT, presenting a high lethality [85]. Pathogenic variants in the *TRPM4* gene, both gain and loss of function, have been identified in patients with different forms of cardiac disorder including conduction defects, BrS and LQTS [77]. The *PKP2* gene, the main gene mutated in arrhythmogenic cardiomyopathy (ACM), has been recently associated with BrS and CPVT. The p.S183N pathogenic variant has been reported in both a patient with BrS and a patient with a definite diagnosis of ACM [221]. However, further studies are needed to establish the pathophysiological mechanisms of these correlations.

### 7.1. SCN5A Clinical Overlap

**Figure 2:**
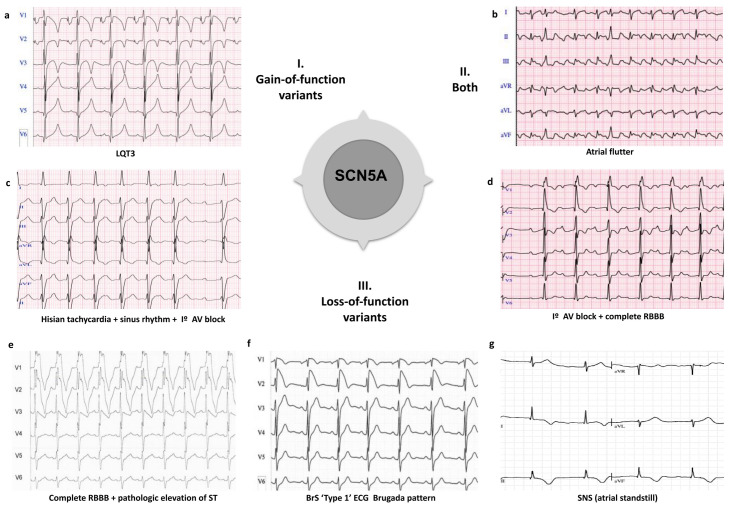
Examples of clinical overlap due to pathogenic variants in the SCN5A gene. I. Phenotype caused by gain-of-function variants in the SCN5A gene: (**a**) LQTS (Long QT syndrome type 3); II. Phenotype caused by both gain-of-function and loss-of-function variants in the SCN5A gene: (**b**) Atrial flutter; III Phenotypes caused by loss-of-function variants in the SCN5A gene: (**c**) Hisian tachycardia + sinus rhythm + I° AV block; (**d**) I° AV block + complete RBBB (right bundle branch block); (**e**) Complete RBBB + pathologic elevation of ST; (**f**) BrS (Brugada syndrome) ‘type 1’ ECG; and (**g**) SNS (sinus node syndrome), atrial standstill.

Pathogenic variants affecting *SCN5A* have been found in all major IAS, as well as in other associated cardiac phenotypes (Figure 2). Gain-of-function pathogenic variants in *SCN5A* (LQT3), have been associated with other arrhythmias including multifocal ectopic Purkinje-related premature contractions [222,223] and atypical CPVT-like phenotype [224]. For instance, p.T1857I and p.I141V variants, have been associated with tachyarrhythmias and exercise-induced polymorphic ventricular arrhythmia [224,225]. However, the correlation between *SCN5A* pathogenic variants and CPVT remains debated. Meanwhile, loss-of-function pathogenic variants in *SCN5A* (usually associated with BrS) have also been implicated in certain phenotypes including isolated cardiac conduction defect and sick sinus syndrome (SSS) [226,227]. In addition, both loss-of-function and gain-of-function pathogenic variants can cause dilated cardiomyopathy (DCM), AF, and overlap syndromes [226,228]. The founder pathogenic variants p.E1784K [229,230], p.F1617del [231], p.1795insD [232], among others, can be manifested as a mixed clinical phenotype of LQTS and/or BrS, even between affected individuals in the same family. Recently, Sasaki, et al. have described the p.A735E pathogenic variant, which could be associated with the overlap of multiple phenotypes in eight carrier individuals, although further studies are needed to ratify this hypothesis [76].

### 7.2. Genetic Overlap of Arrhythmogenic Phenotypes and Epilepsy

Recently, evidence for genetic overlap between IASs and epilepsy has been reported. Sudden unexpected death in epilepsy (SUDEP) share many features with SADS in the young and may have a similar genetic contribution [233,234]. In a systematic review, Anwar and Chahal, et al., found that 11% of the most frequent pathogenic variants identified by molecular autopsy in SUDEP were found in genes related to cardiac channelopathies [235].

### 7.3. Non-Genetic Phenotype Overlapping

Multiple phenotypic overlaps between IASs have been described, in which no underlying genetic cause has been observed ERS and BrS [66,236], BrS and CA [237], ACM and BrS [238]. The overlap of ACM and BrS is controversial, since their diagnostic criteria exclude the coexistence of both syndromes in the same individual. This overlap would not be fully explained by a genetic overlap. The ECG pattern of BrS (drug-induced) was observed both in patients with ACM and pathogenic variants in the *SCN5A* gene [239] as well as in patients without *SCN5A* pathogenic variants [240,241]. Therefore, it is possible that genetic (coding or non-coding variants) and non-genetic (demographic variables or exogenous factors) modifiers could be involved in this variability, all of them contributing additively to the expression of the phenotype. An alternative explanation for phenotypic overlap could be misdiagnosis due to the use of drugs in ECG testing. Notably, it has been shown that flecainide can induce ST-segment elevation in ECG in patients with LQTS3, and lead to misdiagnosis in some cases [242].

## 8. How to Deal with the Variants of Uncertain Significance in Inherited Arrhythmia Syndromes (IASs)

The classification of VUS remains a current challenge in genetic field. When functional and segregation studies are not feasible due to the rarity and exclusivity of some variants, population allele frequency (https://gnomad.broadinstitute.org/ accessed on 3 December 2021) and familial segregation are a fundamental tool for variant classification [132]. Continued reclassification of rare variants based on the ACMG-AMP criteria has led to an increasing understanding of the potential impact of a variant on a disease; for example, it has been shown that rare variants previously described as pathogenic may have too high a population frequency to be responsible for IASs, and that a large number of rare variants may be benign. On the other hand, as mentioned, the ACMG/AMP guidelines present limitations for their application in the classification of rare variants in IASs and their criteria need to be adjusted. To date, few validations have been performed with adjusted criteria. The reasons are probably due to the difficulty of establishing valid and homogeneous criteria that can be generally applied to such heterogeneous diseases such as IAS. Assigning erroneous classifications to variants carries great danger, both for false positives (assigning pathogenic causality to variants that are not) that can have severe consequences, for example leading to the implantation of an unnecessary ICD or, on the contrary, leaving as VUS variants those that are truly causative of the disease. Nevertheless, working with criteria adjusted to each disease could be a useful approach to achieve greater success in the classification of genetic variants in IASs and decrease the number of cases without a conclusive genetic diagnosis. In the recent validation of variants in LQTS and BrS by Walsh et al. they developed a quantitative implementation (disease-specific) of the ACMG-AMP guidelines following the ClinGen recommendations [243]. These refinements consisted of defining population frequency thresholds for rare variants taking into account both disease prevalence and estimated penetrance, together with the maximum allelic contribution. They further used data from case-control studies to identify genetic regions highly enriched in rare variants in IASs cohorts compared to the control population, together with the information derived from functional studies. These data proved to be effective, and implementation of these criteria led to a significant reduction in the proportion of cases with VUS in both syndromes [18].

## 9. Conclusions

Nowadays, the diagnosis, management and risk stratification of SADS in the pediatric population is still a challenge for clinicians. Genetic diagnosis plays an important role in the differential diagnosis of SADS. In addition to directing the clinical management, treatment and risk stratification of patients in most cases, it allows risk stratification and prevention of their relatives, who may remain asymptomatic. International guidelines recommend genetic analysis in families with IASs, testing only the main causal genes associated with each syndrome [244], always in the context of pretest and posttest genetic counseling. For a genetic result of VUS, it is important to emphasize that it does not necessarily imply lower or higher risk for any carrier patient. It means that there is currently insufficient evidence to support or rule out the pathogenic role of the variant in the phenotype. Therefore, clinical translation of VUS should be undertaken with caution and should not be excluded or used in clinical decision-making until follow-up testing is completed, and its clinical role clarified [15]. Continuous refinements in clinical and genetic tools have improved diagnosis in families. Nevertheless, more than 50% of families remain without a conclusive genetic result, with the concern that unexpected death is often the first manifestation of the disease. Owing to recent evaluations of IASs-associated genes according to the evidence-based approach proposed by ClinGen, we now have a better genotype–phenotype correlation of the main causative genes. However, it is necessary to continue this curation process to clarify the association of minority genes with these diseases. Additionally, adjustment of the ACMG-AMP criteria considering the inheritance model, population frequency, prevalence and penetrance of the IASs, among other factors, will allow a more accurate classification of these rare variants before applying knowledge to clinical practice in a personalized approach. 

## Figures and Tables

**Table 1:** Genotype-phenotype IASs correlation. Based on ClinGen [13]. Adapted from Nakajima, et al. [33]. ACM: Arrhythmogenic cardiomyopathy; AE3: Anion exchanger; AF: Atrial fibrillation; AFL: Atrial flutter; AR: autosomal recessive; ATS: Andersen-Tawil syndrome; AVB: atrioventricular block; BrS: Brugada syndrome; CDSP: Systemic primary carnitine deficiency; CPVT: catecholaminergic polymorphic ventricular tachycardia; CND: complex neurodevelopmental disorder; CRDS: Ca^2+^-release deficiency syndrome; DCM: Dilated cardiomyopathy; DD: developmental delay; DM: diabetes mellitus; ERS: Early repolarization syndrome; HCM: cardiomyopathy; Hypertrophic GOF: gain-of-function; ICa: Voltage-gated calcium currents; Ih: Hyperpolarization-activated non-selective cation channels/currents; IK: Delayed rectifier potassium currents; IK-_Ach_: Acetylcholine-activated inward rectifier potassium currents; IK-_ATP_: ATP-sensitive inward rectifier potassium currents; IKr: Rapidly activating IK; IKs: Slowly activating IK; IK1: Inward rectifier potassium currents; INa: Voltage-gated sodium currents; Ito: Transient outward potassium currents; TKOS: triadin knockout syndrome, TS: Timothy syndrome; JLNS: Jervell and Lange–Nielsen syndrome; LOF: loss-of-function; LQTS: Long QT syndrome; LVNC: Left ventricular non-compaction; PCCD: progressive cardiac conduction disease; SCA: Spinocerebellar ataxia; SND: Sinus node dysfunction; SNHL: Sensorineural hearing loss; SQTS: Short QT syndrome; SVT: Supraventricular tachyarrhythmia; WPW: Wolff–Parkinson–White.; XD: X-linked dominant.

Cardiac Phenotype	Inheritance Model	Frequency	Gene Curation	Genes	Main Type of Mutations	Current Affected	Non-Cardiac Phenotype	Phenotypic Overlap (Both LOF/GOF Variants)	Ref.
**LQTS**									
LQT1	AD	30–35%	Definitive genes	*KCNQ1*	LOF	IKs	SNHL (AR), seizures	JLNS (AR), SQTS, AF	[10,33,34,35,36,37,38]
LQT2	AD	25–30%		*KCNH2*	LOF	IKr	Seizures	SQTS, BrS, AF	[10,33,34,39]
LQT3	AD	5–10%		*SCN5A*	GOF	INa	Multiple (including seizures)	BrS, SQTS, CPVT, ERS, AF, AFL, ARVC/D, HCM, DCM, LVNC, SVT, AVB, SND, PCCD, WPW	[10,33,34,40,41,42]
LQT14–16	AD	<1%	Definitive genes with atypical characteristics	*CALM1–3*	GOF	ICa	Seizures, DD	CPVT	[10,33,43,44,45]
LQT17 (TKOS)	AR	<1%		*TRDN*	LOF	ICa	Muscle weakness	CPVT	[10,33,46,47]
LQT5	AD	<1%	Genes with moderate or limited evidence, associated with multiorgan syndromes	*KCNE1*	LOF	IKs	SNHL (AR)	JLNS (AR)	[10,33,34,48]
LQT8	AD	<1%		*CACNA1C*	GOF	ICa	Dysmorphic and neurodevelopmental features	TS, SQTS, BrS, ERS, HCM, AF, SND, CND	[10,33,49,50,51,52,53]
LQT7	AD	<1%		*KCNJ2*	LOF	IK1	Muscle weakness, dysmorphic features, DD, seizures	ATS, SQTS, AF, CPVT, DCM	[10,33,37,42,54,55,56,57,58]
LQTS	AD	<1% each	Other genes with limited evidence	*ANK2* *KCNJ5* *KCNE2* *AKAP9* *SCN4B* *CAV3* *SNTA1*	LOF LOF LOF LOF GOF GOF GOF	Many IK-_Ach_ IKr IKs INa INa INa	Seizures	CPVT, BrS, CND AF, SND AF	[10,33,34,42,48,59,60,61,62,63]
**BrS**									
BrS1	AD	20–30%	Definitive gene	*SCN5A*	LOF	INa	Multiple (including seizures)	BrS, SQTS, CPVT, ERS, AF, AFL, ARVC, HCM, DCM, LVNC, SVT, AVB, SND, PCCD, WPW	[12,64,65,66,67,68,69,70,71,72,73,74,75,76]
BrS	AD	<5%	Genes with moderate or limited evidence	*ABCC9* *ANK2* *KCNH2* *KCNJ8* *KCND3* *KCNE3* *CACNA1C* *CACNB2* *CACNA2D1* *HCN4* *PKP2* *GPD1-L* *TRPM4* *SCN1B–3B SCN10A* *SLMAP RANGRF*	GOF GOF GOF GOF GOF GOF LOF LOF LOF LOF LOF LOF LOF LOF LOF LOF LOF	IK-_ATP_ Many IKr IK-_ATP_ Ito Ito ICa ICa ICa Ih INa INa INa INa INa INa INa	Seizures Seizures Seizures, SCA (See LQT8)	ERS, AF, DCM LQTS, CPVT, CND LQTS, SQTS, AF ERS, AF ERS, AF, CND AF Multiple (see LQT8) SQTS, ERS, CND SQTS, ERS, CND AF, SND ARVC, DCM, ACM, CPVT DM LQTS AF AF	[12,33,37,64,77,78,79,80,81]
	XD			*KCNE5*	LOF	Ito		AF	
**CPVT**									
CPVT1	AD	55–60%	Definitive genes	*RYR2*	GOF	ICa		LQTS, HCM, LVNC, CRDS	[11,48,82,83,84,85,86,87]
CPVT2	AR	3–5%		*CASQ2*	LOF	ICa			[11,48,88]
CPVT3	AR	1–2%		*TECRL*	LOF	ICa			[11,48,89,90]
CPVT4	AD	<1%		*CALM1–3*	LOF	ICa	Seizures, DD	LQTS	[11,43,44,45,48]
CPVT5	AR	1–2%		*TRDN*	LOF	ICa	Muscle weakness	LQTS	[11,47,48,91]
CPVT	AD	<1%	Genes with moderate or limited evidence	*SCN5A* *PKP2* *ANK2* *KCNJ2*	LOF LOF LOF LOF	INa INa Many IK1	Multiple Seizures Seizures (See LQT7)	Multiple (see BrS1) ARVC, DCM, ACM, CPVT LQTS, BrS, CND Multiple (see LQT7)	[11,48,92]
**SQTS**									
SQT1	AD	15%	Definitive gene	*KCNH2*	GOF	IKr	Seizures	LQTS, AF, BrS	[11,33,34,93]
SQT2–3	AD	<5% each	Genes with strong-moderate evidence	*KCNQ1* *KCNJ2*	GOF GOF	IKs IK1	(See LQT1) (See LQT7)	JLNS (AR), SQTS, AF Multiple (see LQT7)	[10,11,33,34,35,36,37,38]
SQTS	AD	<1%	Gene with moderate evidence	*SLC4A3*	LOF	AE3			[11,94]
SQTS	AD AR	<1% each	Genes with limited evidence	*CACNA1C CACNB2* *CACNA2D1* *SCN5A SLC22A5*	LOF LOF LOF LOF LOF	ICa ICa ICa INa INa	(See LQT8) Multiple Metabolic decompensation, skeletal myopathy	Multiple (see LQT8) SQTS, ERS, CND SQTS, ERS, CND Multiple (see BrS1) CDSP	[11,33,65,95,96,97]

## Data Availability

Not applicable.
